# Correction: Prevalence of bacterial coinfection and patterns of antibiotics prescribing in patients with COVID-19: A systematic review and meta-analysis

**DOI:** 10.1371/journal.pone.0307695

**Published:** 2024-07-18

**Authors:** Faisal Salman Alshaikh, Brian Godman, Oula Nawaf Sindi, R. Andrew Seaton, Amanj Kurdi

The affiliation for the fifth author is incorrect. Amanj Kurdi is not affiliated with #4 but with #3: Division of Public Health Pharmacy and Management, School of Pharmacy, Sefako Makgatho Health Sciences University, Pretoria, South Africa.
