# Supplementary material for: Prevalence of bacterial coinfection and patterns of antibiotics prescribing in patients with COVID-19: A systematic review and meta-analysis
Source: PLoS One. 2022 Aug 1;17(8):e0272375. doi: 10.1371/journal.pone.0272375 (PMC9342726; doi:10.1371/journal.pone.0272375)
Supplement: S1 Data — (DOCX) [file pone.0272375.s003.docx]

**Supplementary Data**

*Appendix 1: Key search strategy*

**Table 1: Embase (OVID) Key Search Strategy**

|  | **Searches** | **Results** |
| --- | --- | --- |
| 1 | coronavirus disease 2019.ti,ab,kw. | 25682 |
| 2 | covid-19.ti,ab,kw. | 130651 |
| 3 | severe acute respiratory syndrome coronavirus 2.ti,ab,kw. | 14082 |
| 4 | 1 or 2 or 3 | 134431 |
| 5 | mixed infection.ti,ab,kw. | 4870 |
| 6 | co-infection.ti,ab,kw. | 18590 |
| 7 | 5 or 6 | 23284 |
| 8 | antibiotic agent.ti,ab,kw. | 766 |
| 9 | antimicrobial therapy.ti,ab,kw. | 18563 |
| 10 | antimicrobial activity.ti,ab,kw. | 46766 |
| 11 | antibacterial activity.ti,ab,kw. | 46261 |
| 12 | antiinfective agent.ti,ab,kw. | 31 |
| 13 | 8 or 9 or 10 or 11 or 12 | 103731 |
| 14 | 7 or 13 | 126880 |
| 15 | 4 and 14 | 463 |
| 16 | prevalence/ | 793668 |
| 17 | Observational Studies/ or observational.ti,ab. | 384858 |
| 18 | prospective studies/ or retrospective studies/ or prospective.ti,ab. or retrospective.ti,ab. | 2326072 |
| 19 | randomized controlled trial/ | 665355 |
| 20 | 16 or 17 or 18 or 19 | 3703038 |
| 21 | 15 and 20 | 151 |
| 22 | limit 21 to (human and yr="2019 -Current") | 149 |
| 23 | limit 22 to english language | 147 |

**Table 2: MedLine (OVID) Key Search Strategy**

|  | **Searches** | **Results** |
| --- | --- | --- |
| 1 | COVID-19/ | 88096 |
| 2 | Coronavirus Infections/ | 44949 |
| 3 | SARS-CoV-2/ | 68317 |
| 4 | covid*.ti,ab,kw. | 134027 |
| 5 | coronavirus*.ti,ab,kw. | 68550 |
| 6 | ncov*.ti,ab,kw. | 1606 |
| 7 | sars*.ti,ab,kw. | 60031 |
| 8 | severe acute respiratory syndrome coronavir*.ti,ab,kw. | 16722 |
| 9 | "2019 ncov*".ti,ab,kw. | 1682 |
| 10 | nCOV19*.ti,ab,kw. | 9 |
| 11 | SARSCoV2*.ti,ab,kw. | 45 |
| 12 | "sars cov2*".ti,ab,kw. | 2059 |
| 13 | "coronavirus 19*".ti,ab,kw. | 290 |
| 14 | 1 or 2 or 3 or 4 or 5 or 6 or 7 or 8 or 9 or 10 or 11 or 12 or 13 | 170734 |
| 15 | Coinfection/ | 12379 |
| 16 | coinfect*.ti,ab,kw. | 15898 |
| 17 | co-infect*.ti,ab,kw. | 18471 |
| 18 | "mixed infecti*".ti,ab,kw. | 7718 |
| 19 | "bacterial coinfect*".ti,ab,kw. | 311 |
| 20 | "bacterial co-infect*".ti,ab,kw. | 365 |
| 21 | 15 or 16 or 17 or 18 or 19 or 20 | 44285 |
| 22 | Anti-Bacterial Agents/ | 354528 |
| 23 | Anti-Infective Agents/ | 56119 |
| 24 | antimicrob*.ti,ab,kw. | 184372 |
| 25 | anti-microb*.ti,ab,kw. | 4930 |
| 26 | antiinfect*.ti,ab,kw. | 798 |
| 27 | anti-infect*.ti,ab,kw. | 7984 |
| 28 | antibact*.ti,ab,kw. | 84949 |
| 29 | anti-bact*.ti,ab,kw. | 4701 |
| 30 | antibiot*.ti,ab,kw. | 372008 |
| 31 | 22 or 23 or 24 or 25 or 26 or 27 or 28 or 29 or 30 | 710470 |
| 32 | 21 or 31 | 751355 |
| 33 | 14 and 32 | 4117 |
| 34 | prevalence/ | 311186 |
| 35 | Observational Studies/ or Observational.ti,ab,kw. | 203842 |
| 36 | prospective studies/ or retrospective studies/ or prospective.ti,ab,kw. or retrospective.ti,ab,kw. | 1880121 |
| 37 | randomized controlled trial/ | 535869 |
| 38 | 34 or 35 or 36 or 37 | 2654657 |
| 39 | 33 and 38 | 836 |
| 40 | limit 39 to (english language and humans and yr="2019 - 2022") | 461 |

**Table 3: Cochrane Key Search Strategy**

| **ID** | **Search** | **Hits** |
| --- | --- | --- |
| #1 | MeSH descriptor: [COVID-19] this term only | 398 |
| #2 | MeSH descriptor: [Severe Acute Respiratory Syndrome] this term only | 358 |
| #3 | MeSH descriptor: [SARS-CoV-2] this term only | 294 |
| #4 | (covid*):ti,ab,kw | 5941 |
| #5 | (corona*):ti,ab,kw | 65223 |
| #6 | (coronavirus*):ti,ab,kw | 3372 |
| #7 | (coronavirus infect*):ti,ab,kw | 2051 |
| #8 | ("SARS-Co-V*"):ti,ab,kw | 2144 |
| #9 | ("severe acute respiratory syndrome coronavirus*"):ti,ab,kw | 489 |
| #10 | #1 OR #2 OR #3 OR #4 OR #5 OR #6 OR #7 OR #8 OR #9 | 68000 |
| #11 | MeSH descriptor: [Coinfection] this term only | 210 |
| #12 | MeSH descriptor: [Bacterial Infections] this term only | 3191 |
| #13 | MeSH descriptor: [Respiratory Tract Infections] this term only | 2323 |
| #14 | (coinfect*):ti,ab,kw | 1606 |
| #15 | (co-infect*):ti,ab,kw | 965 |
| #16 | ("co infect*"):ti,ab,kw | 1 |
| #17 | ("mixed infection*"):ti,ab,kw | 579 |
| #18 | ("respiratory infection*"):ti,ab,kw | 1323 |
| #19 | #11 OR #12 OR #13 OR #14 OR #15 OR #16 OR #17 OR #18 | 8180 |
| #20 | MeSH descriptor: [Anti-Bacterial Agents] this term only | 11188 |
| #21 | MeSH descriptor: [Antibiotic Prophylaxis] this term only | 1301 |
| #22 | MeSH descriptor: [Anti-Infective Agents] this term only | 2715 |
| #23 | (antibiotic*):ti,ab,kw | 32360 |
| #24 | (antimicrobial*):ti,ab,kw | 7832 |
| #25 | ("antimicrobial drug*"):ti,ab,kw | 82 |
| #26 | ("antibiotic drug*"):ti,ab,kw | 55 |
| #27 | ("antibiotic agent*"):ti,ab,kw | 2527 |
| #28 | ("empirical antibiotic*"):ti,ab,kw | 166 |
| #29 | ("prophylactic antibiotic*"):ti,ab,kw | 635 |
| #30 | #20 OR #21 OR #22 OR #23 OR #24 OR #25 OR #26 OR #27 OR #28 OR #29 | 42620 |
| #31 | #19 OR #30 | 47991 |
| #32 | #10 AND #31 | 822 |
| #33 | #32 with Cochrane Library publication date Between Nov 2019 and Jun 2021 | 392 |
| #34 | #33 in Cochrane Reviews, Trials | 392 |

**MedRXiv**

Full text or abstract or title "covid-19 AND (Antibiotic OR Coinfection)" (match whole all) and posted between "01 Dec, 2019 and 14 Jun, 2021" Results: **182**
