# Supplementary material for: Prevalence of bacterial coinfection and patterns of antibiotics prescribing in patients with COVID-19: A systematic review and meta-analysis
Source: PLoS One. 2022 Aug 1;17(8):e0272375. doi: 10.1371/journal.pone.0272375 (PMC9342726; doi:10.1371/journal.pone.0272375)
Supplement: Attachment — Submitted filename: PLOS Coinfection Meta-analysis review.docx [file pone.0272375.s004.docx]

Prevalence of Bacterial Coinfection and Patterns of Antibiotics Prescribing in Patients with COVID-19: A Systematic Review and Meta-Analysis.

Manuscript Number: PONE-D-22-06815

**Summary**

The topic of this manuscript is very important. The course of the COVID-19 pandemic remains uncertain and seems increasingly likely it will continue to impact medical systems into the future. What is more certain is the pandemic of antimicrobial resistance (ref). All use of antibiotics contributes to this pandemic, particularly antibiotic use in patients without a bacterial infection. Bacterial coinfection is a real, but very difficult to diagnose complication of viral pneumonia. For this reason, clinicians struggle to determine which patients are likely to benefit from antibiotics. Establishing baseline risk factors for bacterial coinfection in COVID-19 is critical in aiding clinicians to utilize antibiotics in this population. A systematic review and meta-analysis is an excellent tool to determine that risk. The authors perform a systematic review and meta-analysis of the available literature on coinfection in COVID-19. They find that coinfection occurs in about 5% of patients within 48 hours of COVID-19 diagnosis. They also find that antibiotic prescription is over 60% in that same population. They conclude that antibiotics are over prescribed for coinfection in COVID-19.

Major Comments:

While the concept of this study is very important for clinicians and antibiotic stewards, this not unique and is somewhat behind the literature. Multiple meta-analyses and systematic reviews, including one in PLOS ONE, have already been published with findings comparable to this manuscript. In response to these earlier studies, national guidelines statements on antibiotic use in COVID-19 have been published. Currently, clinicians and stewardship programs are advocating more restricted antibiotic use in COVID-19. While these studies have been done, this manuscript is the largest meta-analysis that this reviewer has come across which would serve to cement the literature on incidence of coinfection in COVID-19, validate ongoing antimicrobial stewardship efforts, and affirm clinician’s choices to use less antibiotics in COVID-19.

The authors stratified coinfection and antibiotic use by location. I have not previously seen much literature to suggest the pathophysiology of COVID-19 and coinfection are different between regions. Some regions have different baseline population ages and prevalence of comorbidities which can affect disease severity, and indirectly, risk for coinfection or antibiotic use. What is likely different between regions is robustness of diagnostic modalities for coinfection, availability of various antibiotics, and prevalence of antimicrobial stewardship programs. While some differences exist between the regions, the general message of relatively uncommon coinfection and antibiotic over utilization remains similar between groups. The major weakness of this regional analysis is significant bias towards the United States and Europe. The data are sparce from Asian locations, and there are no studies from Africa or South America. It is difficult to make any conclusions about regional differences given large lack of data from other geographic regions.

Three terms were used in the search. All of these terms are reasonable. This reviewer wonders if they may have picked up more literature if “bacterial,” or “super-infection” were used.

Studies performed in children were not included. It is likely few studies were preformed in children given COVID-19 is less morbid in children. None-the-less, inclusion might have been useful as children are often overlooked in the medical literature and left behind by evidence based medicine.

Non-peer reviewed publications were included in this analysis. This reviewer finds inclusion of non-peer reviewed manuscripts problematic. The rapid push to describe COVID-19 and publish experiences during the pandemic has resulted in many pre-print and non-peer reviewed articles being disseminated. These premature disseminations have resulted in retractions and risk undermining public trust of the medical literature. The authors appropriately list this as a potential limitation of the study later in the manuscript.

The authors chose to only include coinfection occurring within 48 hours of COVID-19 diagnosis. This is helpful as hospital acquired pneumonia occurs after prolonged hospital exposure and is pathophysiologically distinct from community acquired pneumonia.

Diagnosing coinfection is difficult and often done heterogeneously. Diagnosis can be based on clinical factors such as oxygenation, radiography, fever, physical exam findings. Diagnosis can also be aided with laboratory and microbiologic testing, such as inflammatory markers, cultures, and PCR. This is much more difficult in the setting of a pre-existing infection, which can mimic bacterial infection. It would be nice to see a description of how each study defined coinfection. This might possibly explain the high degree of heterogeneity between coinfection rates.

Figure 2 is an easy to read figure clearly demonstrating the finding of a roughly 5% coinfection rate, ranging from 2-10%. This is the figure that most readers will remember as the principle finding of the meta-analysis. This coinfection rate is consistent with rates described by other groups. The variability in rate is understandable given the heterogeneity of the study populations and coinfection diagnosis practices. While the mathematical variability within the 95% confidence interval (2 and 10%) is real, this degree of variability is clinically smaller. When deciding to prescribe antibiotics to a patient, the difference between 2 and 10% prevalence would not be considered significant to most clinicians.

The prevailing organisms causing coinfection are known common causes of pneumonia. It is surprising that E coli was the second most commonly identified organism though. Perhaps this is driven by studies including urinary tract infection (UTI) among their coinfections. If UTIs are driving this finding, it is worth mentioning by the authors, as E coli is an uncommon cause of community acquired pneumonia.

Studying antibiotic use adjacent to coinfection rates is a natural comparison that underlies the importance of determining the true risk of coinfection in COVID-19. It also emphasizes the critical need to effectively identify patients with coinfection and appropriately administer antibiotics. This reviewer is not surprised they found considerable heterogeneity of antibiotic prescribing practices for a number of reasons. Antimicrobial stewardship is performed heterogeneously between hospitals. It is likely that antimicrobial prescription practices were lower in studies performed at hospitals with robust antimicrobial stewardship programs.

funnel plot for antibiotic prescribing practices does not have ideal symmetry. This asymmetry could be attributed to publication bias or heterogeneity of antibiotic prescription practices. A comment to this effect may be warranted.

The authors perform a brief discussion of some of the high impact or outlier studies included in the analysis. This reviewer appreciates this discussion as it lends incite in to the design of included studies. It also lends incite into the high degree of heterogeneity of coinfection literature. One major factor in potential over diagnosis of coinfection is diagnostic culture of non-sterile body sites. The authors comment directly on this challenge in regards to colonization of the urinary track in older diabetic patients as well as colonization of the airways of mechanically ventilated patients.

The authors suggest that there is insufficient evidence for high coinfection rates to justify considerable empiric antibiotic prescribing. This reviewer believes that this manuscript is unable to completely support this statement. While coinfection rates are low, there is a proportion of patients who do have coinfection. It is very likely those patients would have a much worse prognosis if antibiotic administration was delayed. This reviewer takes this manuscript as evidence we need to better identify patients who are unlikely to have a coinfection and avoid using antibiotics in them. Given there is a (small) rate of coinfection, antibiotic use will continue to be necessary in COVID-19, the key is identifying those few patients and sparing antibiotics in the rest.

This study provides the most complete and thorough review and meta-analysis of coinfection and antibiotic prescription practices in COVID-19 to date. Though, the message of the review is not dissimilar to previous systemic reviews and meta-analyses on the topic. That said, the message is abundantly clear, coinfection is a relatively uncommon complication of COVID-19, despite this, antibiotic prescription is very high. This manuscript serves to bolster the ongoing efforts to reduce unnecessary antibiotic prescribing in COVID-19. This study will no doubt serve to support further studies into the timely identification of patients with coinfection and striking an appropriate balance between antibiotic prescription and reducing morbidity and mortality in bacterial coinfections.

Minor Comments:

Line 100: Categorizing information regarding hydroxychloroquine use as “misinformation” strikes this reviewer as problematic and potentially political. There is a growing body of empirical evidence on hydroxychloroquine use in COVID-19. The evidence indicates hydroxychloroquine is not beneficial and likely harmful. Regardless, this study is not the appropriate position to determine what information should be considered “misinformation.”

Line 184: This reviewer is not a trained statistician and unable to comment on the appropriateness of the statistical methods.

Figure 1: Figure is clean and makes understanding study inclusion/exclusion simple.

Table 1: Table is easy to read and compare study similarities and differences.

Figure 3: Sub stratification by region is an interesting aspect of this analysis, this figure demonstrates some of the subtleties between the selected regions

Figure 4: stratifying the findings by study design is good, it shows small benefit of the superior prospective studies, but doesn’t change the message of the manuscript by much.

Figure 5: This figure will but just behind figure 2, in memorability for readers. It contrasts nicely with figure 2, drawing stark contrast between coinfections and antibiotic use.

Line 229: nearly 90% of the patients included in analysis were from two studies. It is likely these studies bear outsized weight on the final outcome of the analysis. The authors recognize this outsized contribution appropriately.

Line 402-406: This reviewer agrees with the hypothesis that coinfection rates are likely linked to diagnostic practice patterns at various hospitals.

Line 420-427: This reviewer agrees with the hypothesis that study design likely influenced coinfection rates owing to more rigorous coinfection definitions in prospective studies.
